# Efficacy and safety of MIL60 compared with bevacizumab in advanced or recurrent non-squamous non-small cell lung cancer: a phase 3 randomized, double-blind study

**DOI:** 10.1016/j.eclinm.2021.101187

**Published:** 2021-11-19

**Authors:** Rui Wan, Xiaorong Dong, Qun Chen, Yan Yu, Shujun Yang, Xiaochun Zhang, Guojun Zhang, Yueyin Pan, Sanyuan Sun, Chengzhi Zhou, Wei Hong, Hui Zhao, Lei Yang, Linian Huang, Rong Wu, Aimin Zang, Rui Ma, Lin Wu, Dongqing Lv, Xiuhua Fu, Jianguo Han, Wenxin Li, Jianchun Duan, Kai Wang, Ou Jiang, Yinglan Chen, Zhongliang Guo, Hongjun Gao, Juyi Wen, Shubin Wang, Enfeng Zhao, Gaofeng Li, Lu Yue, Li Liang, Aiping Zeng, Xiaoshan Wang, Yuxi Zhu, Hongming Pan, Zhaoxia Dai, Weineng Feng, Guofang Zhao, Chuan Lin, Chong Li, Na Li, Yangyi Bao, Yinyin Li, Yanjun Su, Min Zhao, Haohui Fang, Yulong Zhu, Yu Zhang, Lieming Ding, Yang Wang, Xiaobin Yuan, Jie Wang

**Affiliations:** 1 State Key Laboratory of Molecular Oncology, Department of Medical Oncology, National Cancer Center/National Clinical Research Center for Cancer/Cancer Hospital, Chinese Academy of Medical Sciences & Peking Union Medical College, Beijing, China; 2 Cancer Center, Union Hospital, Tongji Medical College, Huazhong University of Science and Technology, Wuhan, China; 3 Medical Oncology, Fuzhou pulmonary Hospital of Fujian, Fuzhou, China; 4 Medical Oncology, Harbin Medical University Cancer Hospital, Harbin, China; 5 Department of Internal Medicine, Henan Cancer Hospital & Affiliated Cancer Hospital of Zhengzhou University, Zhengzhou, China; 6 Cancer Precision Medicine Center, The Affiliated Hospital of Qingdao University, Qingdao, China; 7 Respiratory Department, The First Affiliated Hospital of Zhengzhou University, Zhengzhou, China; 8 Department of Oncology Chemotherapy, the First Affiliated Hospital of USTC (Anhui Provincial Hospital), Hefei, China; 9 Oncology, Xuzhou Central Hospital, Xuzhou, China; 10 Pulonary and Critical Care Medicine, The First Affiliated Hospital of Guangzhou Medical University, Guangzhou, China; 11 Medical Oncology, Zhejiang Cancer Hospital, Hangzhou, China; 12 Respiratory, The Second Hospital of Anhui Medical University, Hefei, China; 13 Department of Respiratory Oncology, Gansu Provincial Cancer Hospital, Lanzhou, China; 14 Department of Respiratory and Critical Care Medicine, The First Affiliated Hospital of Bengbu Medical College, Bengbu, China; 15 Department of Internal Oncology II, Shengjing Hospital of China Medical University, Liaoning, China; 16 Internal Medicine-Oncology, Affiliated Hospital of Hebei, Baoding, China; 17 Medical Oncology Department of Thoracic Cancer (II), Liaoning Cancer Hospital & Institute, Shenyang, China; 18 Thoracic Medicine Department II, Hunan Cancer Hospital, Changsha, China; 19 Respiratory, Taizhou Hospital of Zhejiang Province, Taizhou, China; 20 Department of Respiratory and Critical Care Medicine, The Affiliated Hospital of Inner Mongolia Medical University, Hohhot, China; 21 Oncology, Chifeng Municipal Hospital, Chifeng, China; 22 Oncology, Inner Mongolia People's Hospital, Hohhot, China; 23 Respiratory, The Second Affiliated Hospital Zhejiang University School of Medicine, Hangzhou, China; 24 Department of Oncology, The Second People's Hospital of Neijiang, Neijiang, China; 25 Medical Oncology, Jiangxi Province Cancer Hospital, Nanchang, China; 26 Department of Respiratory Medicine, Shanghai East Hospital, Shanghai, China; 27 Department of pulmonary oncology, The Fifth Medical Center of PLA General Hospital, Beijing, China; 28 Department of Radiation Oncology, The Sixth Medical Center of PLA General Hospital, Beijing, China; 29 Medical Oncology, Peking University Shenzhen Hospital, Shenzhen, China; 30 Three and Four Departments of Radiotherapy and Chemotherapy, Cangzhou Hospital of Integrated Traditional Chinese and Western of Hebei Province, Cangzhou, China; 31 Thoracic surgery, Yunnan Cancer Hospital, Kunming, China; 32 Oncology, Qingdao Municipal Hospital, Qingdao, China; 33 Oncology, Peking University Third Hospital, Beijing, China; 34 Department of Chemotherapy, Affiliated Tumor Hospital of Guangxi Medical University, Nanning, China; 35 Oncology, Sichuan Provincial People's Hospital, Chengdu, China; 36 Department of Oncology, The First Affiliated Hospital, Chongqing Medical University, Chongqing, China; 37 Oncology Department, Sir Run Run Shaw Hospital, Zhejiang University School of Medicine, Hangzhou, China; 38 Department of Thoracic Medical Oncology II, The Second Hospital of Dalian Medical University, Dalian, China; 39 Head and Neck/Thoracic Medical Oncology, The First People's Hospital of Foshan, Foshan, China; 40 Department of Cardiothoracic Surgery, Hwa Mei Hospital, University of Chinese Academy of Sciences (Ningbo No.2 Hospital), Ningbo, China; 41 Oncology, Yibin Second People's Hospital, Yibin, China; 42 Department of Respiratory Medicine, The First People's Hospital of Changzhou, Changzhou, China; 43 Department of Oncology, Suining Central Hospital, Suining, China; 44 Department of Oncology, The First People's Hospital of Hefei, Hefei, China; 45 First Department of Oncology, Shenyang Chest Hospital, Shenyang, China; 46 Department of Pulmonary Oncology, Tianjin Medical University Cancer Institute & Hospital, Tianjin, China; 47 Oncology, Hebei Chest Hospital, Shijiazhuang, China; 48 Respiratory, Anhui Chest Hospital, Hefei, China; 49 Department of Respiratory, Hospital of Traditional Chinese Medicine of Xinjiang Uygur Autonomous Region, Urumqi, China; 50 Respiratory, Nanjing Chest Hospital, Nanjing, China; 51 Betta pharmaceuticals Co., Ltd., Hangzhou, China

**Keywords:** MIL60, biosimilar, bevacizumab, equivalence, non-squamous NSCLC

## Abstract

**Background:**

We compared the efficacy, safety, and immunogenicity of MIL60 with reference bevacizumab as first-line treatment in patients with advanced or recurrent non-squamous non-small cell lung cancer (NSCLC) in this phase 3, randomized, double-blind study.

**Methods:**

Patients with untreated advanced or recurrent NSCLC were randomized (1:1 ratio) to receive either MIL60 or bevacizumab in combination with paclitaxel/carboplatin. Patients with non-progressive disease continued maintenance single-agent MIL60 until disease progression, or intolerable toxicity. The primary endpoint was the 12-week objective response rates (ORR12) by independent review committee (IRC) using RECIST 1.1. Bioequivalence was established if the ORR ratio located between 0.75 and 1/0.75. The trial was registered with clinicaltrials.gov (NCT03196986).

**Findings:**

Between Aug 23, 2017, and May 8, 2019, 517 patients were randomly assigned to MIL60 group (n=257) and bevacizumab group (n=260). In the full analysis set (FAS) population including all randomized and evaluable patients who received at least one dose of MIL60 or bevacizumab, the ORR12 in MIL60 group and bevacizumab group were 48.6% and 43.1%, respectively. The ORR ratio of these two groups were 1.14 (90% CI 0.97-1.33), which fell within the pre-specified equivalence boundaries (0.75-1/0.75). The median DOR was 5.7 months (95% CI 4.5-6.2) for MIL60 and 5.6 months (95% CI 4.3-6.4) for bevacizumab. No significant difference was noted in median PFS (7.2 vs. 8.1 months; HR 1.01, 95% CI 0.78-1.30, p=0.9606) and OS (19.3 vs. 16.3 months; HR 0.81, 95% CI 0.64-1.02, p=0.0755). Safety and tolerability profiles were similar between the two groups. No patient detected positive for Anti-drug antibody (ADA).

**Interpretation:**

The efficacy, safety and immunogenicity of MIL60 were similar with bevacizumab, providing an alternative treatment option for advanced or recurrent non-squamous NSCLC.

**Funding:**

This study was sponsored by Betta Pharmaceutical Co., Ltd.

**Research in Context**

**Evidence before this study**

Bevacizumab was approved in combination with platinum-doublet chemotherapy as first-line treatment for advanced or recurrent non-squamous NSCLC in China in 2015. As of June 2021, we searched PubMed with the terms “bevacizumab”, “biosimilar”, and “NSCLC”. Multiple bevacizumab biosimilars (including ABP 215, QL1101, PF-06439535, IBI305 and SB8, etc.) have been approved in different countries, which are more affordable therapeutic options that can dismantle the cost barrier of reference bevacizumab.

**Added value of this study**

This study aimed to assess the equivalence in efficacy and safety profiles for the bevacizumab biosimilar, MIL60, in Chinese patients with advanced or recurrent non-squamous NSCLC. The findings suggest that the efficacy, safety and immunogenicity of MIL60 were similar with bevacizumab, providing an alternative treatment option for advanced or recurrent non-squamous NSCLC.

**Implications of all available evidence**

Recently, options for bevacizumab therapy in patients with advanced or recurrent non-squamous NSCLC is still worth discussing. The previous research has demonstrated that bevacizumab biosimilars (such as ABP125 and PF-06439535) show similar outcomes in patients mostly from Europe and the USA with advanced or recurrent non-squamous NSCLC. Also, several studies have investigated the use of bevacizumab biosimilars (IBI305 and QL1101) in Chinese population. Herein, we assumed **48%** for the ORR based on the efficacy of bevacizumab in Chinese patients after a symposium with center for drug evaluation (CDE), NMPA, which is higher than that (approximately 38%) in other studies evaluating bevacizumab biosimilars (e.g. ABP 215, PF‑06439535) in western population. Besides, we implemented MIL60 maintenance in this study to further evaluate its safety with a longer exposure. Although direct comparisons of results are difficult because of differences in study design, the median OS (19.3 months) in our study was comparable to that reported with PF-06439535 (19.4 months) and SB8 (14.9 months). This study showed equivalences in efficacy (including ORR, DCR, PFS and OS) and safety profiles when comparing MIL60 with bevacizumab among patients with non-squamous NSCLC in China.

Alt-text: Unlabelled box

## 1 Introduction

Lung cancer is the leading cause of cancer-related deaths worldwide [1]. Non-small cell lung cancer (NSCLC) accounts for 85% of all cases of lung cancer [2]. ECOG1594 trial showed that platinum-based chemotherapy improved median overall survival to 8 months with no significant differences among four chemotherapy regimens [3]. Targeted therapies redefined treatment options for NSCLC with genetic aberrations such as epidermal growth factor (*EGFR*) mutation and anaplastic lymphoma kinase (*ALK*) rearrangement. Cancer immunology enabled the development of immune checkpoint inhibitors that had dramatically altered the therapeutic landscape of patients without those driver genes. Although treatments with chemotherapy, targeted therapy, or immune checkpoint inhibitors lead to tumor shrinkage and durable response time, disease progression inevitably occurs.

Vascular endothelial growth factor (VEGF) acts as a major regulator of angiogenesis in normal and malignant tumor [4]. Increased expression of *VEGF* has been found in most human tumor tissues, including NSCLC, and in many instances, it is associated with increased risks of recurrence, metastasis, and death [5,6]. Bevacizumab (Avastin®) is a recombinant humanized monoclonal antibody that suppresses the biological activity of VEGF and inhibits tumor growth [7]. Combining bevacizumab and platinum-based chemotherapy for patients with recurrent or advanced NSCLC improves progression-free survival (PFS) and overall survival (OS) compared with chemotherapy alone [[8], [9], [10], [11]]. Similarly, EGFR-tyrosine kinase inhibitor (TKI) plus bevacizumab improved PFS compared with EGFR-TKI alone for patients with *EGFR* mutant NSCLC [12].

Bevacizumab was approved in combination with platinum-doublet chemotherapy as first-line treatment for advanced or recurrent non-squamous NSCLC in China in 2015 [8]. Despite all this, patient had limited access to bevacizumab due to various factors such as deficient reimbursement and high costs in China.

Biosimilar is defined as a drug that is similar with an already available biological drug (the reference product) in physical, chemical, and biological aspects [13], and can provide safe and efficacious treatment options for lower costs than the equivalent reference drug. FDA, EMA, as well as China National Medical Products Administration (NMPA) have issued technical guidance and regulatory guidelines, which require the biosimilars show similarity to the reference drug in structure, function, pharmacokinetics, clinical efficacy, and safety [[13], [14], [15], [16]].

MIL60 has similar affinity for VEGF as bevacizumab [17]. High similarity between MIL60 and bevacizumab has been demonstrated with respect to pharmacokinetics (PK), immunogenicity and safety profile in previous study [18]. We conducted this phase 3 study to compare the efficacy and safety of MIL60 plus paclitaxel/carboplatin with bevacizumab plus paclitaxel/carboplatin in the first-line treatment of advanced non-squamous NSCLC.

## 2 Methods

### 2.1 Study design and patients

This phase 3, multicenter, double-blind, parallel, randomized controlled equivalence trial (ClinicalTrials.gov identifier: NCT03196986) was done in 50 centers across China. The study was conducted in compliance with the International Council for Harmonization Good Clinical Practice guidelines, the Declaration of Helsinki and local regulations. The study was reviewed and approved by ethics committees of all participating centers. Written informed consent was obtained from every patient before any study-specific procedures were performed.

Eligible patients were aged between 18 and 75 years with histologically or cytologically confirmed, stage IV or recurrent non-squamous NSCLC with measurable disease according to the Response Evaluation Criteria in Solid Tumors (RECIST) v1.1. The "recurrent" patients refer to those patients with the tumor relapse and metastasis after radical surgery resection. Other inclusion criteria included Eastern Cooperative Oncology Group (ECOG) performance status of 0-1, absence of previous systematic antitumor therapy, adequate bone marrow, hepatic, and renal function, known *EGFR* detection results and a life expectancy >12 weeks. Participants with mixed NSCLC predominantly composed of squamous cell carcinoma or small cell carcinoma were excluded. Exclusion criteria also included bleeding within three months before screening, tumor invasion into large blood vessels, symptomatic central nervous system metastases, known positive *ALK* or *ROS1* translocation, non-healing wounds, ulcers, bone fractures, major surgery within 4 weeks of randomization.

### 2.2 Randomization and masking

All patients were randomly assigned (in a 1:1 ratio) to the MIL60 or bevacizumab groups as an intravenous injection every 3 weeks for 4 to 6 cycles. Randomization was done via stratified-block random method with stratification according to sex (man vs. woman), *EGFR* status (wild-type vs. mutant), and brain metastases (presence vs. absence). A double-blinding technique with in-house blinding was used. Drugs were packaged identically so that the blind was maintained. Investigators, patients, and sponsors who were involved in the treatment or clinical evaluation of the patients were unaware of the treatment assignments.

### 2.3 Procedures

Patients received a maximum of 6 cycles (3 weeks/cycle) of intravenous MIL60 or bevacizumab (15 mg/kg), combined with carboplatin (the area under the curve was 5) and paclitaxel (175 mg/m^2^), followed by MIL60 (7.5 mg/kg) as maintenance therapy. Treatment continued until the following occurred: intolerable toxicity, consent withdrawal, disease progression, loss of follow-up or death. Dose reduction of MIL60 or bevacizumab was not permitted while dose reduction was allowed for paclitaxel and carboplatin followed package insert or local guidelines. Interruption or discontinuation was allowed for MIL60 and bevacizumab for toxicity management.

Tumor imaging was conducted at baseline and thereafter every 6 weeks until progression or unacceptable toxicity, using computed tomography (CT) chest/abdomen/pelvis and magnetic resonance imaging (MRI) brain scans. Disease was assessed by both investigators and the independent review committee (IRC) according to RECIST 1.1. Laboratory tests, vital signs, and physical examinations were conducted on the 1st day of every cycle and at the end of treatment.

For bevacizumab group, the data used for the establishment of population pharmacokinetics (Pop PK) model include 37 healthy male subjects in phase I clinical trial (MIL60-CT01) and 64 patients with advanced or recurrent NSCLC in phase III clinical trial. For MIL60 group, the data of PK model include 39 healthy male subjects in phase I clinical trial and 62 patients with advanced or recurrent NSCLC in phase III clinical trial. Blood samples were collected at baseline, week 1, 4, 7, and 10 from NSCLC patients. For immunogenicity assessments, the anti-drug antibodies (ADAs) were detected at baseline and 4 weeks after the end of treatment. Patients with positive binding ADA were assessed for neutralizing antibodies.

### 2.4 Outcomes

The primary endpoint was objective response rate (ORR_12,_ percentage of patients with complete response [CR] and partial response [PR]) at week 12, as assessed by the IRC using RECIST v1.1. The data cutoff date for analysis of the primary efficacy endpoint was August 1, 2019. Secondary endpoints included ORR_18_, duration of response (DOR), disease control rate (DCR), PFS, OS, and safety. Analyses of DOR, PFS and OS were based on final data after study completion on October 31, 2020. DOR was defined as time from the date of firstly documented objective response (PR or CR) to disease progression. PFS was defined as the time from randomization until the first occurrence of disease progression or death. Tumor response was assessed by the investigator and blinded independent review committee based on RECIST 1.1 criteria.

Safety assessments included measurement of treatment-emergent adverse events (TEAEs), serious adverse events (SAEs), and the proportion of patients who had treatment-related TEAEs. Adverse events were classified and recorded according to the National Cancer Institute Common Terminology Criteria for Adverse Events (CTCAE; v4.03) definitions.

### 2.5 Statistical analysis

Based on the assumption that 48% of patients would achieve ORR_12_ in both MIL60 and bevacizumab groups, a cohort of 225 patients in each group (450 in total) would provide approximate 80% power to confirm the clinical equivalence in ORR_12_ between MIL60 and bevacizumab groups, at a predefined equivalence margin (0.75, 1/0.75) for the 90% CI of the ORR ratio (MIL60/bevacizumab) following NMPA technical guidance on bioequivalence.

Efficacy analyses were done in the full analysis set (FAS) population, which included all patients with target lesion by IRC evaluation who received ≥1 drug dose. Safety analyses were done in all randomly assigned patients who received at least one drug dose.

Clinical equivalence of the primary endpoint was demonstrated by comparing the 2-sided 90% CI of the risk ratio in ORRs between MIL60 and bevacizumab with the prespecified equivalence margin of 0.75−1/0.75. The primary analysis was based on response determined by IRC. Kaplan-Meier analysis was conducted to estimate survival curves. A stratified Cox model was used to estimate the hazard ratios (HRs) and the 95% CI between the two groups. The DCR was analyzed with the same method for ORR. Analysis of DOR included patients in the FAS who had an objective response based on IRC and investigator assessment.

Based on the established Pop PK model, Phoenix NLME was used to simulate the blood concentration of each subject, and Phoenix WinNonlin 8.0 was used to establish non-compartment model for PK parameters in single dose exposure and multiple dose exposure in MIL60 group and bevacizumab group, respectively. PK parameters, including AUC and C_max_ were analyzed by descriptive statistics. We also evaluated the PK similarity by judging whether the 90% CI of the ratio of a log-transformed exposure measure (AUC or C_max_) fell within the range 80-125%.

Adverse events were analyzed by summarizing the number and incidence of TEAEs, SAEs, and treatment-related TEAEs. Immunogenicity was assessed by measuring the proportion of patients who were positive for ADAs and neutralizing antibodies, the titer of ADAs in the plasma of patients, and the change of immunogenicity during the treatment course.

SAS (version 9.4; SAS Institute, Cary, NC, USA) was used for the statistics analysis.

This study adheres to CONSORT guidelines.

### 2.6 Role of funding source

This study was sponsored by Betta Pharmaceutical Co., Ltd., which assisted with data analysis, data interpretation, manuscript preparation and review. All authors had access to the raw data and had final responsibility for the submitted paper.

## 3 Results

### 3.1 Patient characteristics

**Figure 1:**
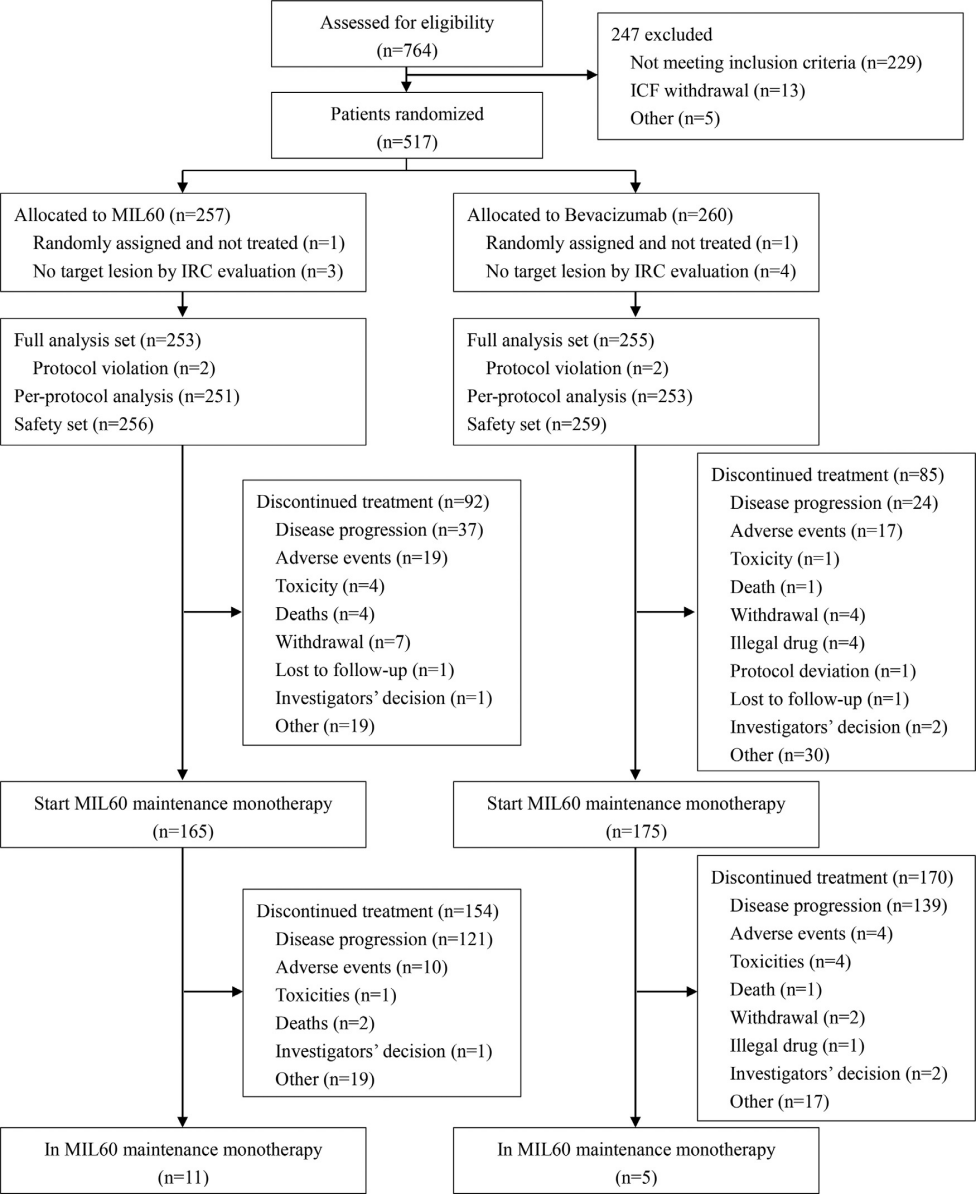
Study flowchart

Between Aug 23, 2017, and May 8, 2019, 764 patients were screened and 517 patients were randomly assigned to MIL60 group (n=257) and bevacizumab group (n=260). Nine patients were excluded from the FAS (2 patients did not receive study drug and 7 patients had no target lesions at baseline by IRC; there were 4 in the MIL60 group and 5 in the bevacizumab group, respectively; Figure 1). Therefore, the FAS population comprised 253 patients in MIL60 group and 255 in bevacizumab group. 515 patients were included in the safety analysis set (SS), 256 and 259 patients in MIL60 and bevacizumab group, respectively. The baseline characteristics were well balanced between groups (Table 1).

**Table 1:** Baseline characteristics

	MIL60 (n=253)	Bevacizumab (n=255)	All patients (n=508)
Age (years)			
Median (IQR, range)	61.0 (13.0; 23-75)	61.0 (11.0; 35-76)	61.0 (12.0; 23-76)
< 60	114 (45.1%)	111 (43.5%)	225 (44.3%)
≥ 60	139 (54.9%)	144 (56.5%)	283 (55.7%)
Sex			
Male	163 (64.4%)	162 (63.5%)	325 (64.0%)
Female	90 (35.6%)	93 (36.5%)	183 (36.0%)
BMI (kg/m²)			
Median (IQR, range)	22.3 (4.1; 15.8-33.8)	22.5 (3.8; 13.9-33.4)	22.4 (4.0; 13.9-33.8)
Body surface area (m^2^)			
Median (IQR, range)	1.6 (0.2; 1.2-2.2)	1.6 (0.2; 1.3 -2.2)	1.6 (0.2; 1.2-2.2)
ECOG performance status			
0	54 (21.3%)	69 (27.1%)	123 (24.2%)
1	199 (78.7%)	186 (72.9%)	385 (75.8%)
*EGFR* status			
Wild-type	199 (78.7%)	198 (77.6%)	397 (78.1%)
Mutant	54 (21.3%)	57 (22.4%)	111 (21.9%)
Brain metastases			
Presence	48 (19.0%)	52 (20.4%)	100 (19.7%)
Absence	205 (81.0%)	203 (79.6%)	408 (80.3%)
Disease stage			
IIIA	1 (0.4%)	2 (0.8%)	3 (0.6%)
IIIB	20 (7.9%)	31 (12.2%)	51 (10.0%)
IV	232 (91.7%)	222 (87.1%)	454 (89.4%)
Smoking status			
Never smoker	127 (50.2)	124 (48.6)	251 (49.4)
Smoker	32 (12.6)	30 (11.8)	62 (12.2)
Former smoker	94 (37.2)	101 (39.6)	195 (38.4)

IQR=interquartile range. Data are number of patients (%) or median (IQR). ECOG=Eastern Cooperative Oncology Group.

During the combination therapy, drugs exposure in two groups was comparable. The median cycle number of treatment was 5.0 (range 1-6) in the MIL60 group and 5.0 (range 1-6) in the bevacizumab group, respectively. There were 111 (43.4%) and 100 (38.6%) patients, completed 6 cycles of combination therapy in the MIL60 and bevacizumab arms, respectively. Median maintenance treatment cycle was 4.0 (range 1-21).

### 3.2 Efficacy

**Figure 2:**
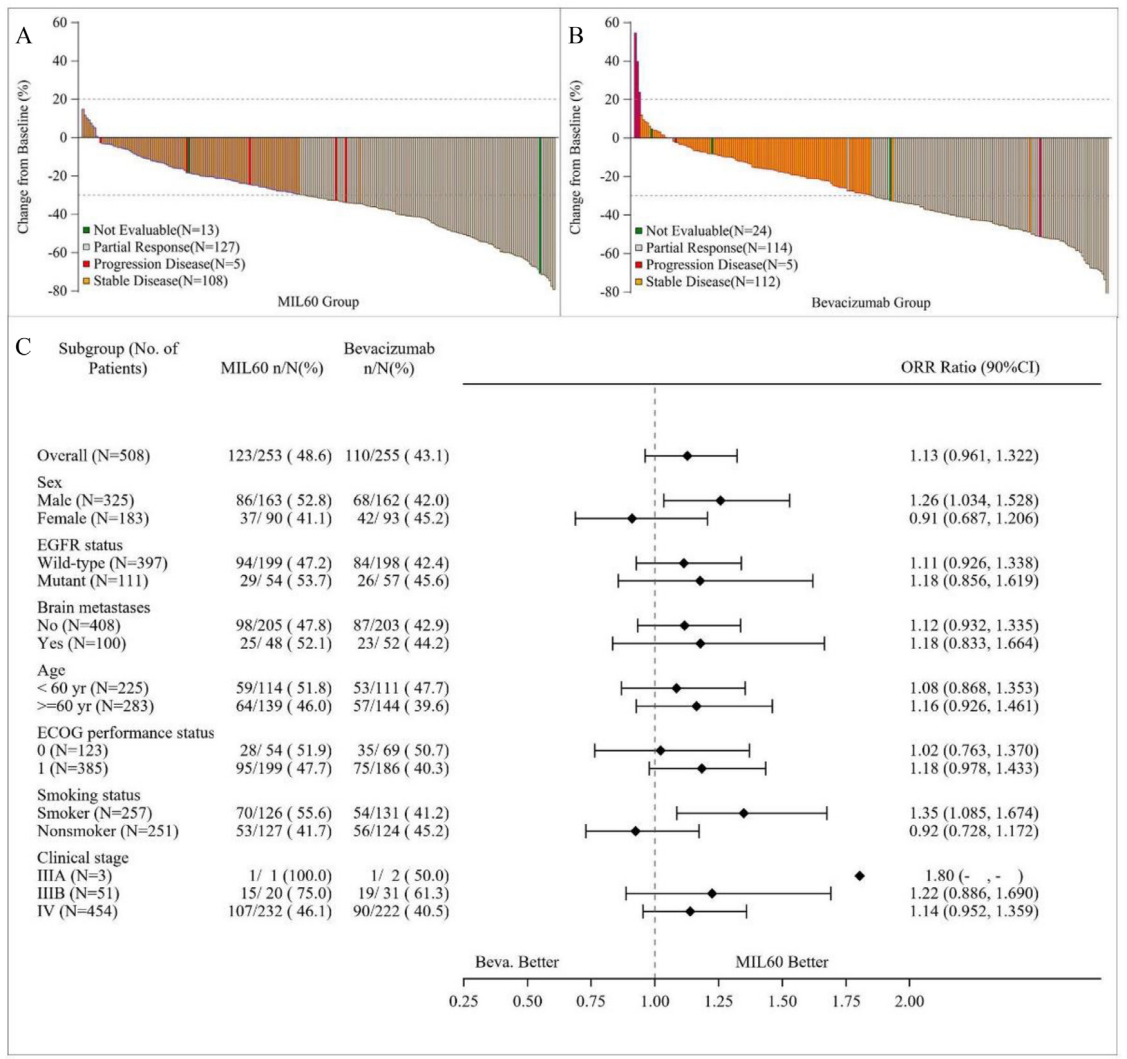
Waterfall plot of best percent change in target lesions from baseline in MIL60 arm (A) and bevacizumab (B), and subgroup analysis for tumor response (C). Data cutoff date was Aug 1, 2019.

In FAS, ORR_12_ assessed by IRC were 48.6% (95% CI 42.6-57.8) and 43.1% (37.4-52.1) in MIL60 and bevacizumab group, respectively (Table 2). The risk ratio of MIL60 to bevacizumab for ORR_12_ was 1.14 (90% CI 0.97-1.33), which fell within the equivalent boundary values specified in the protocol (0.75, 1/0.75). The best percent change from baseline in size of target lesions for patients with measurable disease of the two groups was shown in Figure 2A and 2B. The investigator-assessed ORR_12_ also fell within the equivalent boundary values (**Table S1**). The IRC-assessed ORR_18_ was 50.2% (95% CI 43.7-58.8) in the MIL60 group and 44.7% (95% CI 38.4-53.2) bevacizumab group, respectively (**Table S2**). The investigator-assessed ORR_18_ was in accordance with IRC-assessed ORR_18_ (**Table S3**). The DCRs of the MIL60 and bevacizumab groups determined by IRC were 92.9% (95% CI 88.4-97.1) and 88.6% (95% CI 83.4-92.4), respectively (**Table S4**). In an analysis of response according to patient characteristics (Figure 2**C**), ORR ratios were within the predefined equivalence margins regardless of age (<60 or ≥60 years), *EGFR* status (wild-type or mutant), ECOG PS (0 or 1), clinical stage (IIIB or IV) or brain metastases (presence or absence).

**Table 2:** Best tumor response

	MIL60 (n=253)	Bevacizumab (n=255)
Complete response (CR)	0	0
Partial response (PR)	123 (48.6)	110 (43.1)
Stable disease (SD)	112 (44.3)	116 (45.5)
Progressive disease (PD)	5 (2.0)	5 (2.0)
Unevaluable	13 (5.1)	24 (9.4)
12-week Objective response rate (ORR_12_)	123 (48.6%)	110 (43.1%)
95% CI	42.6-57.8	37.4-52.1
Treatment comparison (vs. bevacizumab group)		
Stratified ORR risk ratio*	1.14	
90% CI of risk ratio*	0.97-1.33	

Data cutoff date was Aug 1, 2019. Data are n (%). ORR defined as the percentage of patients within each treatment group who achieved complete response or partial response with RECIST version 1.1. *Based on generalized linear model (GLM) with stratification variables.

**Figure 3:**
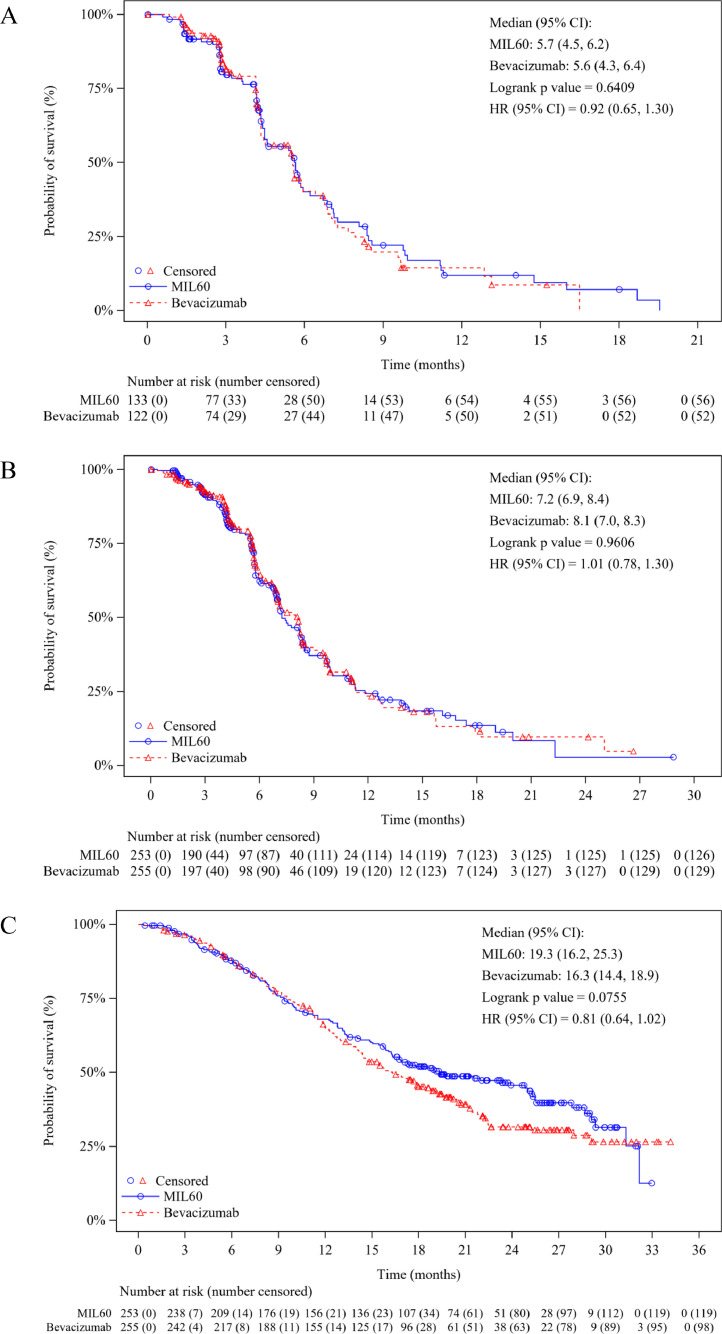
Kaplan–Meier plot of duration of response (A), progression-free survival (B) and overall survival (C) in the MIL60 and reference bevacizumab groups as assessed by IRC. Data cutoff date was October 31, 2020.

At the extended data cutoff for survival (October 31, 2020), the median follow-up for PFS was 8.2 months (95% CI 6.7-8.6), 180 (71.1%) patients in the MIL60 group and 176 (69%) in the bevacizumab group had PFS events (161 progression and 19 deaths in MIL60, 163 progression and 13 deaths in bevacizumab). The IRC-assessed median DOR was 5.7 months (95% CI 4.5-6.2) for MIL60 and 5.6 months (95% CI 4.3-6.4) for bevacizumab, respectively (Figure 3A). The median PFS was 7.2 months (95% CI 6.9-8.4) for MIL60 and 8.1 months (95% CI 7.0-8.3) months for bevacizumab (HR 1.01, 95% CI 0.78-1.30, *p*=0.9606; Figure 3B). The median DOR and PFS assessed by investigators were consistent with those assessed by IRC (**Figure S1 and S2**). 134 (53.0%) patients in the MIL60 group and 157 (61.6%) in the bevacizumab group had OS events, the median overall survival was 19.3 months (95% CI 16.2-25.3) for MIL60 and 16.3 months (95% CI 14.4-18.9) for bevacizumab, respectively (HR 0.81, 95% CI 0.64-1.02, *p*=0.0755; Figure 3C). Moreover, the treatments after progression were summarized in **Table S5**.

### 3.3 Safety

No statistically significant difference was discovered between the MIL60 and bevacizumab groups in the incidence of TEAEs (99.6% vs. 98.8%, 95% CI -0.7-2.3), grade 3 or higher TEAEs (70.3% vs. 72.6%, 95% CI -9.1-4.7), SAEs (28.1% vs. 28.6%, 95% CI -8.2-7.3), or treatment-related TEAEs (78.9% vs. 81.1%, 95% CI -9.1-4.7) (**Table S6**).

Grade 3 or higher TEAEs occurred in 180 (70.3%) patients in the MIL60 group and 188 (72.6%) in the bevacizumab group. The most common grade ≥3 treatment-related TEAEs in both groups were neutropenia, leucopenia, hypertension, bone marrow suppression and febrile neutropenia (Table 3). Common treatment-related SAEs in the MIL60 group and bevacizumab group included febrile neutropenia, thrombocytopenia, pulmonary infection, bone marrow suppression, neutropenia, leukopenia, and anemia (**Table S7**).

**Table 3:** Common treatment-related TEAEs (Safety set)

	MIL60 (n=256)		Bevacizumab (n=259)	
	All grade	Grade 3-5	All grade	Grade 3-5
Neutropenia	65 (25.4%)	44 (17.2%)	57 (22.0%)	42 (16.2%)
Leucopenia	63 (24.6%)	27 (10.5%)	65 (25.1%)	27 (10.4%)
Anaemia	57 (22.3%)	0	59 (22.8%)	0
Proteinuria	50 (19.5%)	0	37 (14.3%)	0
Thrombocytopenia	45 (17.6%)	0	34 (13.1%)	0
Fatigue	34 (13.3%)	0	33 (12.7%)	0
Hypertension	31 (12.1%)	15 (5.9%)	31 (12.0%)	11 (4.2%)
Nausea	25 (9.8%)	0	24 (9.3%)	0
Decreased appetite	21 (8.2%)	0	23 (8.9%)	0
Epistaxis	17 (6.6%)	0	20 (7.7%)	0
Lymphocytopenia	15 (5.9%)	0	13 (5.0%)	0
Abnormal liver function	14 (5.5%)	0	10 (3.9%)	0
Diarrhoea	12 (4.7%)	0	19 (7.3%)	0
Hematuria	12 (4.7%)	0	16 (6.2%)	0
Vomiting	11 (4.3%)	0	19 (7.3%)	0
Febrile neutropenia	8 (3.1%)	8 (3.1%)	13 (5.0%)	13 (5.0%)

Data are n (%).The table shows all treatment-related adverse events that occurred in 5% or more of patients.

Discontinuation was reported in 6 patients (2 in the MIL60 group and 4 in the bevacizumab group) due to MIL60/bevacizumab-related toxicities, including sudden cardiac death and cerebral hemorrhage for MIL60, and heart failure, pulmonary infection, upper gastrointestinal bleeding, gastrointestinal perforation, and febrile neutropenia for bevacizumab. Eleven deaths were related to study treatment, including 5 (one cerebral hemorrhage, two sudden deaths, one respiratory failure and sudden cardiac death) and 5 (one heart failure, one upper gastrointestinal bleeding, one gastrointestinal perforation, one sudden death, one febrile neutropenia and one pulmonary infection) patients in the MIL60 and bevacizumab groups, respectively.

A total of 515 patients were analyzed for ADAs. No patient had positive result during treatment.

### 3.4 Population pharmacokinetic analysis

Final model parameters were estimated with acceptable precision (**Table S8-S10**). In bevacizumab group, the volume of distribution in the central compartment (V), volume of distribution in the peripheral compartment (V2), systemic clearance in the central compartment (Cl) and systemic clearance in the peripheral compartment (Cl2) were 2.94 L, 4.82 L, 0.00665 L/h, and 0.0242 L/h, respectively. The single dose exposure AUC_0-t_, AUC_0-∞_ and C_max_ were 56900 ± 12900 h*μg/mL, 101000 ± 31500 h*μg/mL and 330 ± 62.0 μg/mL, while for the steady-state exposure, the values of AUC_0-tau,_ C_max,ss_ and C_avg,ss_ were 116000 ± 30900 h*μg/mL, 465 ± 94.0 μg/mL and 230 ± 61.2μg/mL, respectively. The values of V, V2, Cl and Cl2 in MIL60 group were 2.95 L, 2.14 L, 0.00814 L/h, and 0.0215 L/h, respectively. The single dose exposure AUC_0-t_, AUC_0-∞_ and C_max_ were 61600 ± 12500 h*μg/mL, 102000 ± 25200 h*μg/mL and 313 ± 62.2 μg/mL, respectively. The values of AUC_0-tau,_ C_max,ss_ and C_avg,ss_ in the steady-state exposure were 113000 ± 21700 h*μg/mL, 447 ± 75.3 μg/mL and 223 ± 43.0 μg/mL, respectively. In single and steady state, 90% CI of geometric mean ratio of above exposure (AUC and C_max_) in MIL60 group was between 80% and 125% compared with bevacizumab group.

In the final models, gender, body weight, health status and albumin were all identified as covariates of the effective influence. The covariates affecting the CL were sex and body weight, while the covariates affecting the V2 were health status and albumin. CL increased with the elevation of body weight and was higher in males, and V2 decreased with the elevation of albumin and was lower in healthy subjects.

The final model was bootstrapped to evaluate the stability and there were no significant differences in Cl or V2 between the MIL60 and bevacizumab in NSCLC patients. The PK parameters also showed that there was no significant difference in the exposure of MIL60 and bevacizumab in patients with advanced or recurrent NSCLC.

## 4 Discussion

In this study, we established the therapeutic equivalence between MIL60 and bevacizumab when combined with paclitaxel and carboplatin in the first-line treatment for advanced non-squamous NSCLC. The 90% CI of the difference in ORR_12_ risk ratio met the pre-specified equivalence boundaries. Efficacy equivalence between MIL60 and bevacizumab was also supported by sensitivity analyses and all secondary outcomes. Moreover, we found no significant difference for safety, Pop PK, or immunogenicity between two groups.

Despite vital roles in exploring clinical benefits for novel anticancer agents, survival-based endpoints are unsuitable for demonstrating biosimilar [19]. Considering inconsistent treatment effects on survival in lung cancer in several studies, a more sensitive endpoint, ORR, which is purely attributable to the treatment, is chosen for comparative clinical studies as the primary endpoint; besides, it is difficult to estimate the treatment-effect size of bevacizumab using either progression-free survival or overall survival as the primary endpoint due to a paucity in the number of available studies with overall survival or progression-free survival as the primary endpoint [20]. For NMPA, equivalence was considered established if a 90% CI of the ORR risk ratio fell within 0.75-1/0.75. The clinical equivalence between the MIL60 and bevacizumab was confirmed by an ORR risk ratio of 1.14 (90% CI: 0.97-1.33), within the predefined equivalence margin of 0.75 to 1/0.75. Similarly, the clinical equivalence was confirmed by an ORR ratio either between the IBI305 and bevacizumab (0.95, 90% CI: 0.803 to 1.135) or between the QL1101 and bevacizumab (0.93, 90% CI: 0.8 to 1.131), within the predefined equivalence margin of 0.75 to 1.33 [21,22]. Notably, previous studies using an assumed ORR of approximately 38% [21,23], we assumed 48% for the ORR based on a symposium with center for drug evaluation (CDE), NMPA, which better fitted the characteristics of Chinese population. Although cross-trial comparisons should be cautious, ORR in the ECOG 4599 treated with bevacizumab plus paclitaxel/carboplatin was 35%, and was therefore similar with the response rates observed in the current study (48.6% in the MIL60 group and 43.1% in the bevacizumab group). Furthermore, ORR in Chinese patient population was 54% in bevacizumab plus paclitaxel/carboplatin based on the phase III BEYOND trial [8].

For survival-based endpoints, the median PFS in ECOG 4599 study treated with bevacizumab plus paclitaxel/carboplatin was 6.2 months [3], which were shorter than those observed in both groups in this study (7.2 months in MIL60 group and 8.1 months in bevacizumab group). The results observed in the MIL60 group were also comparable to previous studies of bevacizumab, including the BEYOND study (median PFS 9.2 months) [8], AVAiL study Asian subgroup (median PFS, 8.2 months) [10,24], and SAiL study Asian subgroup (median PFS 8.8 months) [25], which further confirmed the clinical equivalence of MIL60 with bevacizumab. Despite some similarities, this study included some patients with asymptomatic CNS metastases and *EGFR* mutation. MIL60 showed therapeutic equivalence to bevacizumab regardless of the presence of CNS metastases, in the asymptomatic CNS metastases subgroup, the ORR_12_ ratio evaluated by IRC was 1.12 (90% CI: 0.93-1.34), while in the CNS metastases negative subgroup, the ORR_12_ ratio assessed by IRC was 1.18 (90% CI: 0.83-1.66). Moreover, patients with *EGFR* mutation were also eligible in our study, which was different with those in IBI305 study [7]. Subgroup analysis demonstrated that no significant difference in the effects of biosimilars among the patients with wild-type or mutant *EGFR* status.

The median OS in both arms in our study (19.3 months in MIL60 group and 16.3 months in bevacizumab group) seems longer than that in the ECOG 4599 study arm treated with bevacizumab; however, this should be interpreted with caution due to the improvements in toxicity management, supportive care and subsequent lines of therapy for patients with advanced NSCLC. The survival time in MIL60 group was slightly longer than that in bevacizumab group, but there was no significant difference. This may be due to the fact that all patients in bevacizumab group crossed to MIL60 group for maintenance treatment after the combination therapy. Although direct comparisons of results are difficult because of differences in study design, the median OS in our study was comparable to that reported with PF-06439535 (19.4 months) and SB8 (14.9 months) [26,27].

The frequency, profile, and severity of AEs were comparable between MIL60 and bevacizumab. Moreover, the incidence of AEs commonly associated with anti-VEGF toxicities was comparable between groups. Immunogenicity was similar, and no patients developed binding ADAs in either group and no patients developed neutralizing antibodies. This finding has important clinical implications because biosimilars are usually approved across all indications for the originator product, and these anti-VEGF toxicities are common across bevacizumab indications. MIL60 maintenance was implemented in both groups in this study for patients with responsive or stable disease. A total of 326 patients received maintenance MIL60 with a median cycle of 4.0 (range 1-21). MIL60 maintenance therapy was well tolerated without significant toxicities. 28 (8.6%) patients had grade ≥3 MIL60-related AEs and 23 (7.1%) patients had SAEs. Besides, 2 (0.6%) patients discontinued study treatment including coma (0.3%) and dyspnea (0.3%), and 13 (4.0%) patients required dose interruption due to TEAEs. The safety results of maintenance therapy were similar with those of combined therapy.

Although Pop PK analyses have been previously reported for biosimilars in patients [28] and healthy subjects [29,30], little was based on a comparative clinical study in patients with cancer except PF-06439535 [26]. In the present study, population PK analysis identified baseline body weight, sex and albumin as significant covariates influencing both CL and *V*_2_, similar with previous bevacizumab results [31,32].

Although the combination of bevacizumab and chemotherapy improves the therapeutic efficacy, the high cost and insufficient medical insurance support are known barriers to comprehensive adoption of bevacizumab in clinical practice [33]. The addition of bevacizumab to the paclitaxel/carboplatin for NSCLC leads to an incremental cost-effectiveness ratio of $299,155 per quality-adjusted life year in China, which notably exceeds the accepted Chinese society willingness-to-pay level of $23,970 [34]. General, the discounts of biosimilar drugs were 20%-35% of their reference products in European union and the price of biosimilars was reported as 60% lower than reference products in China [35]. Hence the development of effective and safe biosimilars will provide greater access for patients and will lower costs for these life-saving treatments. So far, several bevacizumab biosimilars have been approved by FDA and EMA, including ABP 215 (Amgen Inc., Thousand Oaks, California, USA), PF‑06439535 (Pfizer, Groton, Connecticut, USA). In China, QL1101 (Qilu Pharmaceutical, Jinan, China) and IBI305 (Innovent Biologics, Inc., Suzhou, China) have been approved by NMPA, and several other potential biosimilars will come onto market in the future. The development of biosimilars provides the public with greater access to treatment options, and could lower health-care costs through competition and increase access to life-saving drugs.

A limitation of this study is the lack of comparison between maintenance MIL60 and bevacizumab. However, the focus of this study was on the therapeutic equivalence of MIL60 and bevacizumab when combined with chemotherapy. Additionally, to the best of our knowledge, randomized studies of maintenance therapy were little in biosimilars development, and it is difficult to determine pre-specified equivalence criteria during a maintenance phase. Therefore, we only evaluated the efficacy and safety of MIL60 maintenance in this study. Besides, intention to treat population was not the main analysis population. Considering the ORR as the primary endpoint, FAS population, due to with target lesion and receiving ≥1 drug dose can more accurately reflect the treatment effect of study drug. Although the combination of carboplatin/paclitaxel/bevacizumab is no longer the standard-of-care for the first-line treatment of patients with advanced non-squamous NSCLC, these results could still be useful, given that: i) there are some settings in which immunotherapy is not available; ii) there are patients that have a contra-indication to immunotherapy and iii) that bevacizumab can be used in combination with chemotherapy and immunotherapy.

In conclusion, the present multicenter, randomized, phase 3 study provides strong evidence of the clinical equivalence of MIL60 to bevacizumab in terms of efficacy, safety, Pop PK and immunogenicity. MIL60 provides a cost-effective alternative treatment for patients with non-squamous NSCLC.

## Funding

This study was sponsored by Betta Pharmaceutical Co., Ltd., which assisted with data analysis, data interpretation, manuscript preparation and review. All authors had access to the raw data and had final responsibility for the submitted paper.

## Contributors

The principal investigator (JW) designed the trial with the sponsor. R Wan, YW and XBY wrote the manuscript. R Wan, XRD, QC, YY, SJY, XCZ, GJZ, YYP, SYS, CZZ, WH, HZ, L Yang, LNH, R Wu, AMZ, RM, LW, DQL, XHF, JGH, WXL, JCD, KW, OJ, YLC, ZLG, HJG, JYW, SBW, EFZ, GFL, L Yue, LL, APZ, XSW, YXZ, HMP, ZXD, WNF, GFZ, C Lin, C Li, NL, YYB, YYL, YJS, MZ, HHF, YLZ, YZ recruited patients and analyzed and interpreted data. LMD and YW were involved in the study conduct and supervision. R Wan and JW accessed and were responsible for the raw data associated with the study. All authors reviewed and approved the final version of the manuscript.

## Data sharing statement

The sponsor (Betta Pharmaceuticals Co., Ltd.) will provide access to individual de-identified participant data from the present study, upon request by qualified researchers whose proposals are approved. Betta will also consider requests for the protocol and statistical analysis plan. Requests for access to the patient-level data from this study can be submitted via email to corresponding author and proposals are required to be attached for approval.

## Declaration of Competing Interest

Lieming Ding, Yang Wang and Xiaobin Yuan are employees of Betta Pharmaceuticals, which provided funding for the study. Other authors declared no conflict of interests.

## Appendix Supplementary materials

Image, application 1
