# Supplementary material for: Efficacy and safety of MIL60 compared with bevacizumab in advanced or recurrent non-squamous non-small cell lung cancer: a phase 3 randomized, double-blind study
Source: eClinicalMedicine. 2021 Nov 19;42:101187. doi: 10.1016/j.eclinm.2021.101187 (PMC8606331; doi:10.1016/j.eclinm.2021.101187)
Supplement: Supplementary file 1 [file mmc1.docx]

**Table S1. ORR achieved by week 12 assessed by investigator (FAS)**

|  | **MIL60 (n=253)** | **Bevacizumab (n=255)** |
| --- | --- | --- |
| Complete response (CR) | 0 | 1 (0.4%) |
| Partial response (PR) | 125 (49.4%) | 115 (45.1%) |
| Stable disease (SD) | 98 (38.7%) | 107 (42.0%) |
| Progressive disease (PD) | 15 (5.9%) | 8 (3.1%) |
| Unevaluable | 15 (5.9%) | 24 (9.4%) |
| 12-week Objective response rate (ORR_12_) | 125 (49.4%) | 116 (45.5%) |
| 95% CI of ORR_12_ | 43.232-55.583 | 39.364-51.617 |
| Treatment comparison (vs bevacizumab group) | | |
| Stratified ORR risk ratio^*^ | 1.08 |  |
| 90% CI of risk ratio^*^ | 0.93-1.26 |  |

Data cutoff date was Aug 1, 2019. Data are n (%). ORR defined as the percentage of patients within each treatment group who achieved complete response or partial response at week 12 accordance with RECIST version 1.1. ^*^Based on generalized linear model (GLM) with stratification variables.

**Table S2. ORR achieved by week 18 assessed by IRC (FAS)**

|  | **MIL60 (n=253)** | **Bevacizumab (n=255)** |
| --- | --- | --- |
| Complete response (CR) | 0 | 0 |
| Partial response (PR) | 127 (50.2%) | 114 (44.7%) |
| Stable disease (SD) | 108 (42.7%) | 112 (43.9%) |
| Progressive disease (PD) | 5 (2.0%) | 5 (2.0%) |
| Unevaluable | 13 (5.1%) | 24 (9.4%) |
| 18-week objective response rate (ORR_18_) | 127 (50.2%) | 114 (44.7%) |
| 95% CI of difference | 43.657, 58.788 | 38.412, 53.193 |
| Treatment comparison (vs bevacizumab group) | | |
| Stratified ORR risk ratio^*^ | 1.13 |  |
| 90% CI of risk ratio^*^ | 0.97, 1.32 |  |

Data cutoff date was Aug 1, 2019. Data are n (%). ORR defined as the percentage of patients within each treatment group who achieved complete response or partial response at week 12 accordance with RECIST version 1.1. ^*^Based on generalized linear model (GLM) with stratification variables.

**Table S3. ORR achieved by week 18 assessed by investigator (FAS)**

|  | **MIL60 (n=253)** | **Bevacizumab (n=255)** |
| --- | --- | --- |
| Complete response (CR) | 0 | 1 (0.4%) |
| Partial response (PR) | 134 (53.0%) | 123 (48.2%) |
| Stable disease (SD) | 89 (35.2%) | 99 (38.8%) |
| Progressive disease (PD) | 15 (5.9%) | 8 (3.1%) |
| Unevaluable | 15 (5.9%) | 24 (9.4%) |
| 12-week Objective response rate (ORR_12_) | 134 (53.0%) | 124 (48.6%) |
| 95% CI of ORR_18_ | 46.799-59.129 | 42.478-54.777 |
| Treatment comparison (vs bevacizumab group) | | |
| Stratified ORR risk ratio^*^ | 1.09 |  |
| 90% CI of risk ratio^*^ | 0.94-1.26 |  |

Data cutoff date was Aug 1, 2019. Data are n (%). ORR defined as the percentage of patients within each treatment group who achieved complete response or partial response at week 18 accordance with RECIST version 1.1. ^*^Based on generalized linear model (GLM) without stratification variables.

**Table S4. DCR assessed by IRC and investigator (FAS)**

|  | **IRC assessment** | | **Investigator assessment** | |
| --- | --- | --- | --- | --- |
|  | **MIL60 (n=253)** | **Bevacizumab (n=255)** | **MIL60 (n=253)** | **Bevacizumab (n=255)** |
| Complete response (CR) | 0 | 0 | 2 (0.8%) | 1 (0.4%) |
| Partial response (PR) | 127 (50.2%) | 114 (44.7%) | 136 (53.8%) | 127 (49.8%) |
| Stable disease (SD) | 108 (42.7%) | 112 (43.9%) | 85 (33.6%) | 95 (37.3%) |
| Progressive disease (PD) | 5 (2.0%) | 5 (2.0%) | 15 (5.9%) | 8 (3.1%) |
| Unevaluable | 13 (5.1%) | 24 (9.4%) | 15 (5.9%) | 24 (9.4%) |
| Disease control rate (DCR) | 235 (92.9%) | 226 (88.6%) | 223 (88.1%) | 223 (87.5%) |
| 95% CI of DCR | 88.4-97.1 | 83.4-92.4 | 82.1-92.4 | 83.4 -91.5 |
| Treatment comparison (vs bevacizumab group) | | |  |  |
| Stratified DCR risk ratio^*^ | 1.05 |  | 1.00 |  |
| 90% CI of risk ratio^*^ | 1.01-1.10 |  | 0.95-1.06 |  |

Data cutoff date was Aug 1, 2019. Data are n (%). The DCR was evaluated as the overall efficacy, which was different from the ORR_12_ data at cutoff data. ^*^Based on generalized linear model (GLM) with stratification variables.

**Table S5. Treatments after progression.**

|  | MIL60  (n=131) | Bevacizumab (n=122) | Total (n=253) |
| --- | --- | --- | --- |
| Chemotherapy | 74 (56.5) | 73 (59.8) | 147 (58.1) |
| Anlotinib | 32 (24.4) | 24 (19.7) | 56 (22.1) |
| Radiotherapy | 29 (22.1) | 18 (14.8) | 47 (18.6) |
| PD-1 monoclonal antibody | 16 (12.2) | 20 (16.4) | 36 (14.2) |
| Icotinib | 14 (10.7) | 12 (9.8) | 26 (10.3) |
| Gefitinib | 13 (9.9) | 15 (12.3) | 28 (11.1) |
| Bevacizumab | 10 (7.6) | 17 (13.9) | 27 (10.7) |
| Osimertinib | 7 (5.3) | 4 (3.3) | 11 (4.3) |
| Afatinib | 4 (3.1) | 3 (2.5) | 7 (2.8) |
| Cabozantinib | 3 (2.3) | 0 | 3 (1.2) |
| Erlotinib | 3 (2.3) | 0 | 3 (1.2) |
| Crizotinib | 2 (1.5) | 4 (3.3) | 6 (2.4) |
| Apatinib | 1 (0.8) | 5 (4.1) | 6 (2.4) |
| Lenvatinib | 1 (0.8) | 0 | 1 (0.4) |
| Sunitinib | 1 (0.8) | 0 | 1 (0.4) |
| TQB3139 | 1 (0.8) | 1 (0.8) | 2 (0.8) |
| Vandetanib | 1 (0.8) | 0 | 1 (0.4) |
| Alectinib | 0 | 3 (2.5) | 3 (1.2) |
| AL8326 | 0 | 1 (0.8) | 1 (0.4) |

**Table S6. Overall summary of treatment-emergent adverse events (TEAEs)**

| **TEAE type** | **MIL60 (n=256)** | **Bevacizumab (n=259)** | **Difference (MIL60/ Bevacizumab) (95% CI)** |
| --- | --- | --- | --- |
| Any TEAEs | 255 (99.6%) | 256 (98.8%) | 0.8 (-0.7, 2.3) |
| Treatment related TEAEs | 202 (78.9%) | 210 (81.1%) | -2.2  (-9.1, 4.7) |
| TEAE grade ≥3 | 180 (70.3%) | 188 (72.6%) | -2.3  (-10.1, 5.5) |
| Any SAEs | 72 (28.1%) | 74 (28.6%) | -0.4  (-8.2, 7.3) |
| Fatal TEAEs | 5 (2.0%) | 5 (1.9%) | 0.0  (-2.4, 2.4) |
| TEAEs leading to treatment termination | 5 (2.0%) | 5 (1.9%) | 0.0  (-2.4, 2.4) |
| TEAEs leading to transient discontinuation of MIL60/bevacizumab | 65 (25.4%) | 64 (24.7%) | 0.7  (-6.8, 8.2) |
| TEAEs leading to transient discontinuation of chemotherapy | 59 (23.0%) | 67 (25.9%) | -2.8  (-10.2, 4.6) |
| AEs leading to permanent discontinuation of MIL60/bevacizumab | 19 (7.4%) | 15 (5.8%) | 1.6  (-2.7, 5.9) |
| AEs leading to permanent discontinuation of chemotherapy | 20 (7.8%) | 17 (6.6%) | 1.2  (-3.2, 5.7) |
| AESIs | 124 (48.4%) | 128 (49.4%) | -1.0  (-9.6, 7.7) |

Data are n (%). Only TEAEs are summarized. For each type, patients are included only once, even if they had multiple events in that category. SAE, serious adverse event; AESIs, adverse events of special interest.

**Table S7. Common treatment-related SAEs (SS population)**

|  | **MIL60 (n=256)** | **Bevacizumab (n=259)** |
| --- | --- | --- |
| Febrile neutropenia | 6 (2.3 %) | 12 (4.6%) |
| Thrombocytopenia | 6 (2.3%) | 3 (1.2%) |
| Pulmonary infection | 6 (2.3%) | 3 (1.2%) |
| Bone marrow failure | 5 (2.0%) | 11 (4.2%) |
| Neutropenia | 4 (1.6%) | 4 (1.5%) |
| Leukopenia | 3 (1.2%) | 5 (1.9%) |
| Anaemia | 0 (0%) | 4 (1.5%) |

Data are n (%).

**Table S8. Parameter estimates and confidence intervals from the final model and bootstrap analysis for MIL60 and bevacizumab**

|  |  |  |  | **Bootstrap** | | |  |
| --- | --- | --- | --- | --- | --- | --- | --- |
| Group | Parameters | Estimate | SD | Median | 95% CI Lower | 95% CI Upper | Bias(%) |
| Bevacizumab | V (L) | 2.94 | 0.0661 | 2.93 | 2.74 | 3.09 | -0.36 |
|  | V2 (L) | 4.82 | 0.496 | 4.68 | 3.67 | 5.92 | -3.07 |
|  | Cl (L/h) | 0.00665 | 0.000537 | 0.00671 | 0.00532 | 0.00799 | 0.90 |
|  | Cl2 (L/h) | 0.0242 | 0.00172 | 0.0239 | 0.0203 | 0.0283 | -1.27 |
|  | Gender effect on Cl | 0.276 | 0.0724 | 0.263 | 0.102 | 0.463 | -4.47 |
|  | Weight effect on V | 0.371 | 0.116 | 0.342 | -0.403 | 0.851 | -7.76 |
|  | Health effect on V2 | -0.996 | 0.112 | -0.959 | -1.24 | -0.721 | -3.67 |
|  | stdev0 | 0.115 | 0.0177 | 0.112 | 0.0804 | 0.153 | -2.56 |
| MIL60 | V (L) | 2.95 | 0.0638 | 2.95 | 2.82 | 3.09 | 0.04 |
|  | V2 (L) | 2.14 | 0.114 | 2.14 | 1.85 | 2.62 | 0.21 |
|  | Cl (L/h) | 0.00814 | 0.000243 | 0.00814 | 0.00756 | 0.00862 | -0.02 |
|  | Cl2 (L/h) | 0.0215 | 0.00164 | 0.0216 | 0.0190 | 0.0256 | 0.71 |
|  | ALBU effect on V2 | -3.00 | 0.346 | -2.95 | -3.66 | -2.13 | -1.77 |
|  | Weight effect on Cl | 0.735 | 0.152 | 0.737 | 0.359 | 1.04 | 0.33 |
|  | stdev0 | 0.0949 | 0.00734 | 0.0940 | 0.0815 | 0.109 | -0.91 |

Bias% = (Median – Estimate) / Estimate * 100. Cl=systemic clearance in the central compartment. Cl2=systemic clearance in the peripheral compartment. SD=standard deviation. V=volume of distribution in the central compartment. V2=volume of distribution in the peripheral compartment. ALBU=albumin.

**Table S9. PK parameters in single dose exposure of MIL60 and bevacizumab**

| **Parameters** | **MIL60 (n=62)** | **Bevacizumab (n=64)** |
| --- | --- | --- |
| Dose (mg) | 938 (158) | 954 (166) |
| λ_z_ (1/h) | 0.00183 (0.00056) | 0.00166 (0.00068) |
| T_1/2_ (h) | 411 (110) | 475 (147) |
| T_max_ (h) | 2.03 (0.50) | 2.00 (0.50) |
| C_max_ (μg/mL) | 313 (62.2) | 330 (62.0) |
| AUC_0-t_ (h*μg/mL) | 61600 (12500) | 56900 (12900) |
| AUC_0-∞_ (h*μg/mL) | 102000 (25200) | 101000 (31500) |
| Vz (mL) | 5450 (1300) | 6500 (1640) |
| CL (mL/h) | 9.57 (2.25) | 10.1 (2.72) |
| Treatment comparison (vs bevacizumab group) | | |
| λ_z_ (1/h) | 113.52% | 90% CI 103.15-124.93 |
| T_1/2_ (h) | 88.09% | 90% CI 80.04-96.94 |
| T_max_ (h) | 101.66% | 90% CI 94.23-109.68 |
| C_max_ (μg/mL) | 94.74% | 90% CI 89.30-100.51 |
| AUC_0-t_ (h*μg/mL) | 108.66% | 90% CI 101.89-115.87 |
| AUC_0-∞_ (h*μg/mL) | 102.49% | 90% CI 94.72-110.91 |
| Vz (mL) | 84.62% | 90% CI 78.49-91.22 |
| CL (mL/h) | 96.06% | 90% CI 89.05-103.62 |

Data are mean (SD).

**Table S10. PK parameters in steady-state exposure of MIL60 and bevacizumab**

| **Parameters** | **MIL60 (n=62)** | **Bevacizumab (n=64)** |
| --- | --- | --- |
| Dose (mg) | 932 (159) | 943 (176) |
| λ_z_ (1/h) | 0.00167 (0.000574) | 0.00118 (0.000321) |
| T_1/2_ (h) | 462 (157) | 624 (150) |
| T_max_ (h) | 0.750 (0.00) | 0.773 (0.132) |
| C_max, ss_ (μg/mL) | 447 (75.3) | 465 (94.0) |
| AUC_0-∞_ (h*μg/mL) | 212000 (80700) | 265000 (108000) |
| C_min, ss_ (μg/mL) | 134 (39.5) | 151 (47.5) |
| C_tau, ss_ (μg/mL) | 138 (42.3) | 158 (51.5) |
| C_avg, ss_ (μg/mL) | 223 (43.0) | 230 (61.2) |
| CL (mL/h) | 8.55 (2.08) | 8.51 (1.97) |
| Vz (mL) | 5370 (1150) | 7420 (1520) |
| AUC_0-tau_ (h*μg/mL) | 113000 (21700) | 116000 (30900) |
| Treatment comparison (vs bevacizumab group) | | |
| λ_z_ (1/h) | 138.47% | 90% CI 126.96-151.02 |
| T_1/2_ (h) | 72.22% | 90% CI 66.22-78.77 |
| T_max_ (h) | 97.86% | 90% CI 95.39-100.39 |
| C_max, ss_ (μg/mL) | 96.79% | 90% CI 91.45-102.44 |
| AUC_0-∞_ (h*μg/mL) | 80.95% | 90% CI 72.68-90.16 |
| C_min, ss_ (μg/mL) | 89.63% | 90% CI 81.74-98.30 |
| C_tau, ss_ (μg/mL) | 87.66% | 90% CI 79.68-96.45 |
| C_avg, ss_ (μg/mL) | 98.92% | 90% CI 92.24-106.07 |
| CL_ss_ (mL/h) | 100.32% | 90% CI 93.51-107.64 |
| Vz (mL) | 72.45% | 90% CI 67.86-77.35 |
| AUC_0-tau_ (h*μg/mL) | 98.92% | 90% CI 92.24-106.07 |

Data are mean (SD).

**Figure S1.** **Kaplan–Meier plot of duration of response assessed by investigators.**


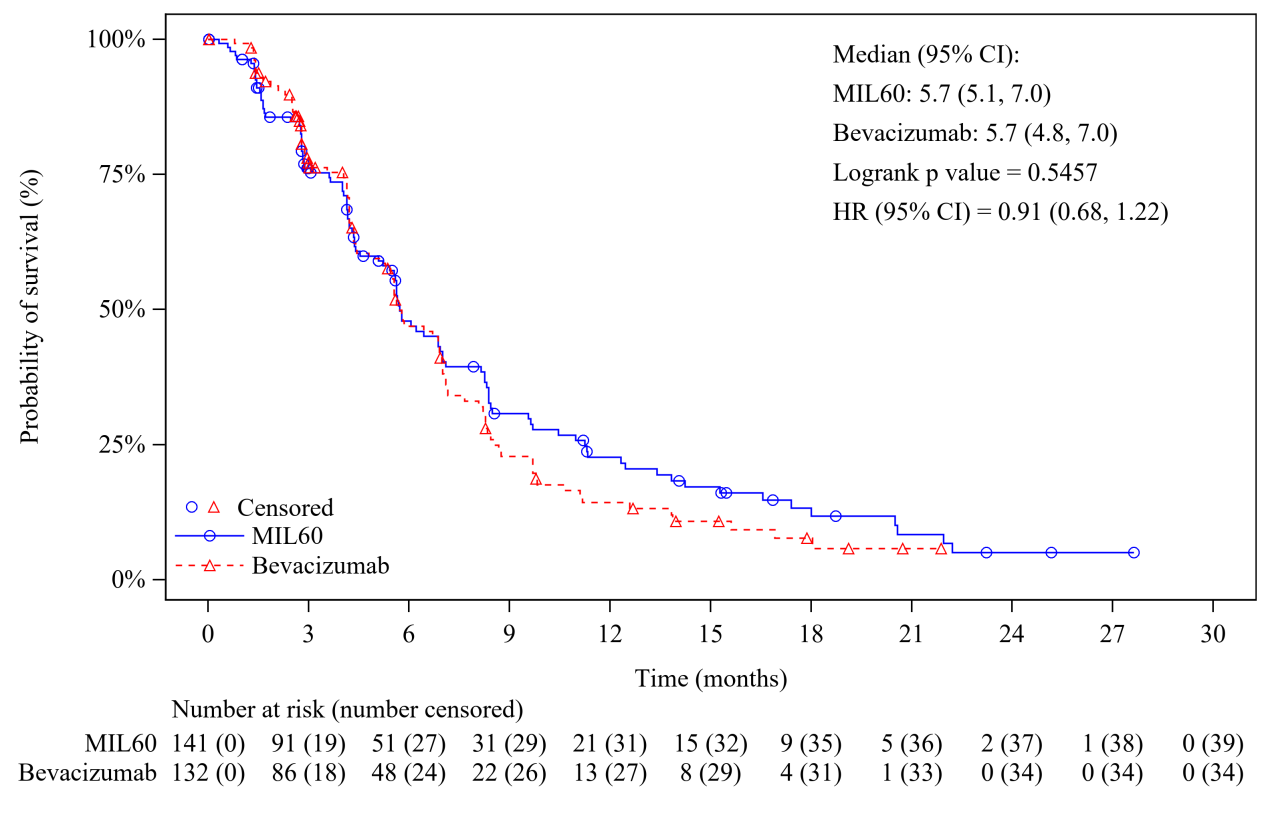


Data cutoff date was October 31, 2020.

**Figure S2.** **Kaplan–Meier plot of progression-free survival assessed by investigators.**


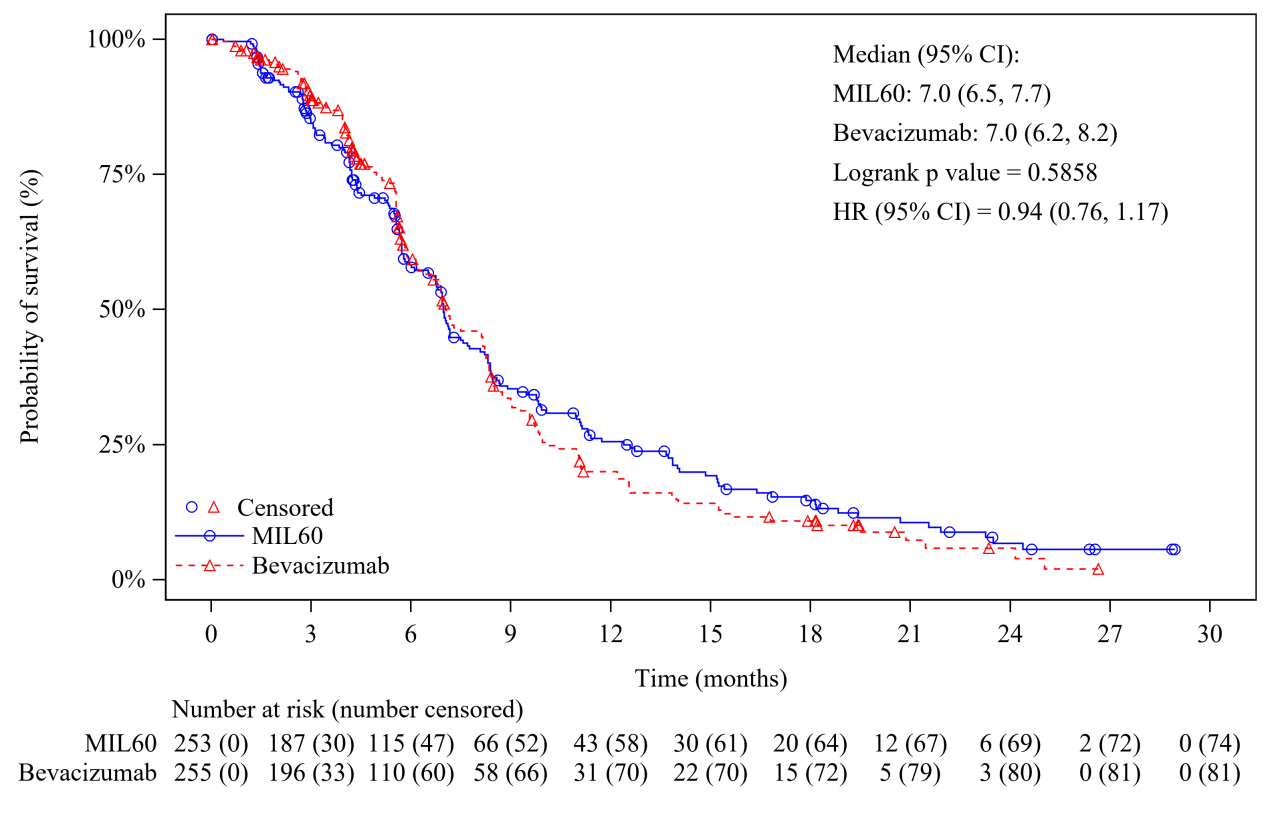


Data cutoff date was October 31, 2020.
